# Supplementary material for: Wanting what hurts: D1 dopamine neuronal stimulation in CeA is sufficient to induce maladaptive attraction
Source: Commun Biol. 2025 Nov 17;8:1574. doi: 10.1038/s42003-025-08944-6 (PMC12624019; doi:10.1038/s42003-025-08944-6)
Supplement: Supplementary file 1 — Supplementary information [file 42003_2025_8944_MOESM1_ESM.pdf]

Supplementary Information

Wanting what hurts: D1 dopamine neuronal stimulation in CeA is sufficient to induce maladaptive attraction

Nguyen and Berridge

Supplementary Note 1: CeA laser self-stimulation

hSyn ChR2. Six of 19 hSyn ChR2 rats met a >50 illuminations criteria for high self-stimulation on Day 1 (earning 299.5 ±52.89 illuminations), and 3 hSyn ChR2 rats met criteria for moderate self-stimulation (i.e., earning between 10 – 50 illuminations; averaging 21.67 ±5.49 illuminations). The remaining 10 hSyn ChR2 rats were classed as failures to self-stimulate, earning fewer than 10 illuminations on Day 1. On days 2 and 3, 6 additional hSyn ChR2 rats rose to meet the >50 illumination criteria for high self-stimulation (total of 12 rats earning 648.67 ±247.45 illuminations), while 7 hSyn ChR2 rats remained failures to self-stimulate. Self-stimulation did not differ between laser parameter groups (high intensity: 473 nm, 10 mW, 40 Hz; $N = 4$, 255.67 ±164.32; low intensity:473 nm, 3 mW, 25 Hz; $N = 15$, 256.51, ±105.53) across the three test days ($t_{17} = 0.004$, $p = 1$).

Males (152.94 ±66.87, $N = 11$) and females (424.54 ±183.83, $N = 8$) did not differ in self-stimulation ($t_{17} = 1.56$, $p = 0.14$).

D1 ChR2. Eight of 16 D1 ChR2 rats met the ≥50 criteria for high self-stimulation on Day 1, (earning 836.38 ±279.38 illuminations), 6 others met the criteria for moderate self-stimulation (≥10 - <50 illuminations; earning 25 ±7.04 illuminations), and the 4 remaining D1 ChR2 failed to meet any criteria for laser self-stimulation on Day 1. On Day 2, 7 rats met the criteria for high self-stimulation (earning 1285 ±430.03 illuminations), 6 rats met the criteria for moderate self-stimulation (earning 19.5 ±1.82 illuminations), and 3 rats failed to self-stimulate. On Day 3, 10 rats met criteria for high self-stimulation (earning 1310.1 ±496.23 illuminations), 2 rats met criteria for moderate self-stimulation (earning 29 ±9 illuminations), and 4 D1 ChR2 rats remained failures to self-stimulate.

There was no sex difference detected between self-stimulation by male (477.14 ±300.79, $N = 7$) or female rats D1 ChR2 (708.19 ±387.34, $N = 9$), and they did not differ in number of active port/spout responses ($t_{14} = 0.45$, $p = 0.66$).

A2 ChR2. Among A2(D2) ChR2 rats, all rats failed to meet any self-stimulation criteria on any day. Across the 3 days, the A2(D2) ChR2 rats actually made more inactive (2 ±0.19) than active (1.22 ±0.22) responses ($t_2 = 6.95$, $p = 0.02$), suggesting if anything, an avoidance of CeA A2(D2) neuronal excitation (473 nm, 10 mW, 40 Hz) (Figure 1e).

CRF ChR2. Among CRF ChR2 rats, only 1 of 9 rats met the high self-stimulation criteria on Day 1 (earning 60 illuminations; 473 nm, 10 mW, 40 Hz), 4 CRF ChR2 rats met the moderate self-stimulation criteria (earning 20.75 ±8.79), and 4 CRF ChR2 rats failed to self-stimulate. On Days 2 and 3, no CRF ChR2 rats met the high self-stimulation criteria, 6 CRF ChR2 rats met the moderate self-stimulation criteria (earning 24.75 ±7.33 illuminations on Day 2 and 22 ±5.17 on Day 3), and the remaining 3 CRF ChR2 rats continued to fail to self-stimulate. However, as a group, CRF ChR2 did not significantly self-stimulate overall, in terms of more active versus inactive responses across all 3 days ($t_8 = 0.96$, $p = 0.366$) (Figure 1f). Thus, it appears that some

CRF ChR2 individual rats do self-stimulate CeA laser at mostly moderate levels, but other individuals fail to self-stimulate at all, giving only marginal performance to the group as a whole. This is largely consistent with previous reports (Baumgartner et al., 2021; 2022).

Supplementary Note 2: Shock rod

Control eYFP. No sex differences were detected, as male (1.78 ±0.41, $N = 12$) and female rats (1.71 ±0.32, $N = 8$) did not differ in number of shock rod contacts ($t_{18} = 0.12$, $p = 0.91$).

hSyn ChR2. Both the 10 mW; 40 Hz and the 3 mW; 25 Hz laser configurations produced similar shock rod attraction, and the number of shocks received did not differ between them ($t_{24} = 0.86$, $p = 0.4$), indicating maladaptive attraction to be a robust CeA ChR2 phenomenon across more than one stimulation configuration. No sex difference in shock rod attraction was detected, as similar number of shock rod contacts were made by both males (13.95 ±1.71, $N = 15$) and females (13.85 ±1.76, $N = 11$), and the two sexes did not differ in shock rod contacts across the three days ($t_{24} = 0.04$, $p = 0.97$).

Further, 17 out of 26 hSyn ChR2 rats reached the maximum-allowed number of 20 shocks before the end of a session on at least one of the three test days, and 10 of these hSyn ChR2 rats reached the maximum of 20 shocks on all three test days, and so were removed early from the shock rod chamber before the end of the 20 min session on each of these days (otherwise they may have received additional shocks)

Among the hSyn ChR2 rats that did self-stimulate, approximately 80% reached the maximum allowed 20 shock rod contacts in at least one test session ($N = 10$), and 60% reached 20 contacts in all three sessions ($N = 7$). Among the rats that failed to self-stimulate, approximately 60% reached 20 contacts in at least one test session ($N = 4$), while 30% reached 20 contacts in all three sessions ($N = 2$).

*D1 ChR2***.** Both the 10 mW; 40 Hz and 3 mW; 25 Hz laser stimulation configurations produced similar shock rod attraction in D1 ChR2 rats and the two configurations did not differ in number of shocks received ($t_{17} = 1.96$, $p = 0.07$). Again, males (14.71 ±2, $N = 7$) and females (14.31 ±1.88, $N = 12$) made similar numbers of shock rod contacts across the three days, and there was no sex difference ($t_{17} = 1.96$, $p = 0.07$).

Among D1 ChR2 rats that did self-stimulate, approximately 80% reached 20 shock rod contacts in at least one test session ($N = 10$), and 30% reached 20 contacts in all three sessions ($N = 4$). Among D1 ChR2 rats that failed to self-stimulate, approximately 75% reached 20 shock rod contacts in at least one test session ($N = 3$), and 25% reached 20 contacts in all three sessions ($N = 1$)

Supplementary Figure 1: Different behavioral phenotypes of rod contacts

For both hSyn ChR2 rats and D1 ChR2 rats, shock rod contacts were made either with paw, snout, or as nibbles or bites with mouth and teeth. Paw touches and snout touches were typically both brief <1 sec contacts with a forepaw, snout, or mouth and teeth, usually followed immediately by a reflexive flinch and rapid drawback of the paw or head. However, in oral nibble/bite contacts, the rat closed its teeth onto the rod, and sometimes followed with repeated chewing motions that usually lasted >1 sec, finally ending when the rat voluntarily disengaged or involuntarily flinched away from the shock rod in reaction to shock.

Although hSyn ChR2 and D1 ChR2 rats were similar in overall number of shocks received, they differed in the detail of behavioral patterns used to touch the shock rod as revealed by a microanalysis of video recorded behavior. Over 77% of contacts by hSyn ChR2 rats were with paw or snout (39.67 ±2,82 of 52.75 ±3.56 total contacts across days 1-3) (79% paw; 21% snout). Only 23% of hSyn ChR2 contacts were oral made by mouth, tongue or teeth (13.08 ±2.4), and all of these were of short duration (1.17 ±0.67 sec). By comparison, D1 ChR2 rats made nearly 200% more oral contacts than hSyn rats, so that 47% of total D1 ChR2 contacts were with mouth, tongue or teeth (25.45 ±4.54 of 51.27 ±3.62; D1 ChR2 vs hSyn ChR2: $t_{21} = 2.47$, $p = 0.022$). Further, D1 ChR2 oral contacts trended towards being twice as long than hSyn ChR2 oral contacts, averaging 2.2 ±0.67 sec in duration, though that difference did not reach significance ($t_{12} = 1.43$, $p = 0.18$). Yet D1 ChR2 rats still made 53% snout or paw contacts (25.82 ±2.87) (69% paw; 31% snout; D1 ChR2 vs. hSyn ChR2: $t_{21} = 3.44$, $p = 0.002$) (Figure 3a).

The hSyn ChR2 individuals that did self-stimulate made more oral contacts (18.33 ±2.56) than hSyn ChR2 individuals that failed to self-stimulate (7.6 ±3.41; $t_9 = 2.56$, $p = 0.03$), but made similar numbers of paw/snout contacts ($t_9 = 0.03$, $p = 0.98$) (Figure 3d). Similarly, D1 ChR2 individuals that did self-stimulate made numerically more oral contacts (30 ±5.07) than D1 ChR2 individuals that failed to self-stimulate (13.33 ±10), but the difference was not statistically significant ($t_9 = 1.81$, $p = 0.1$). D1 ChR2 rats that did self-stimulate made numerically 30% fewer paw/snout contacts (23.13 ±3.37) than D1 ChR2 rats that failed to self-stimulate (33 ±1), but the difference was also not statistically significant ($t_9 = 1.67$, $p = 0.13$) (Figure 3e). Overall, we conclude that individuals that showed laser self-stimulation displayed more oral interactions on the shock rod than individuals that failed to self-stimulate laser, especially with hSyn ChR2 activation, similar to a previous report that self-stimulation facilitated longer oral contacts[1].

Microstructure of attraction: D1 ChR2 vs hSyn ChR2 behavioral patterns. Although D1 ChR2 rats and hSyn ChR2 rats displayed similar overall intensity of attraction to the laser-paired shock rod in terms of number of contacts and shocks received, there were detectable differences in the behavioral microstructure or detailed movement patterns the two groups used to touch the shock rod. For example, D1 ChR2 rats often chewed on the shock rod despite receiving oral shocks and

made twice as many oral contacts with mouth or teeth (44%) as did hSyn ChR2 rats (≤20%). Instead hSyn ChR2 rats made ≥80% of shock rod contacts with their forepaws, compared to 56% by D1 ChR2 rats. Conceivably, the higher rate of oral contacts from CeA D1 ChR2 excitation may be related to ingestive roles previously reported for CeA D1 neurons in promoting eating[2,3].

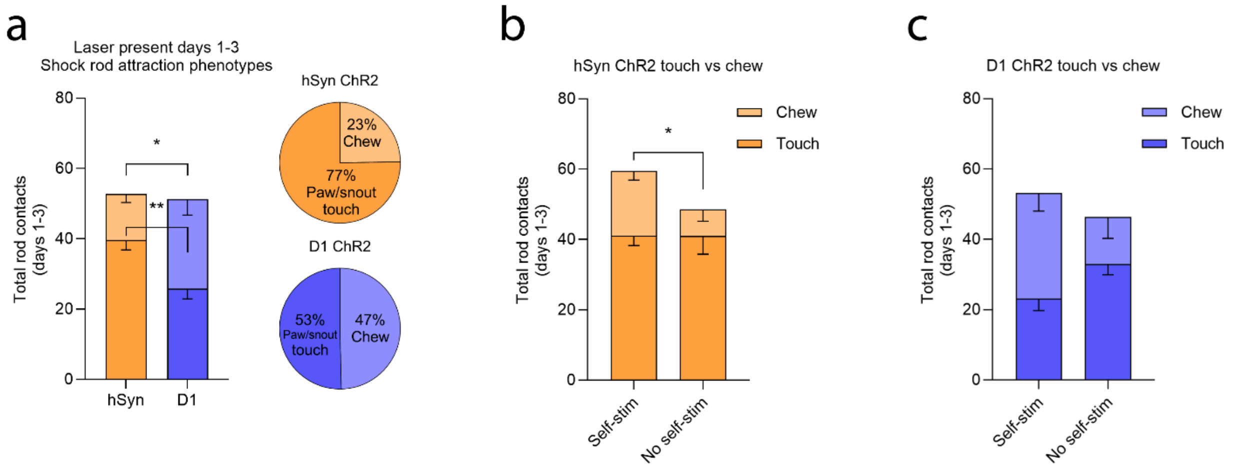

Supplementary Figure 1. a. CeA hSyn ($N = 14$) and D1 ChR2 ($N = 15$) rats differed in behavioral microstructure of shock-rod contacts. The hSyn ChR2 rats made mostly paw/snout contacts, whereas D1 ChR2 rats made relatively more oral chewing contacts. **b.** hSyn ChR2 rats that self-stimulate ($N = 6$) made more oral contacts on the shock rod than hSyn ChR2 rats that don't self-stimulate ($N = 5$). **c.** D1 ChR2 rats that self-stimulate ($N = 8$) made more oral contacts on the shock rod than D1 ChR2 rats that failed to self-stimulate ($N = 3$), but the difference was not statistically significant.

Supplementary Figure 2

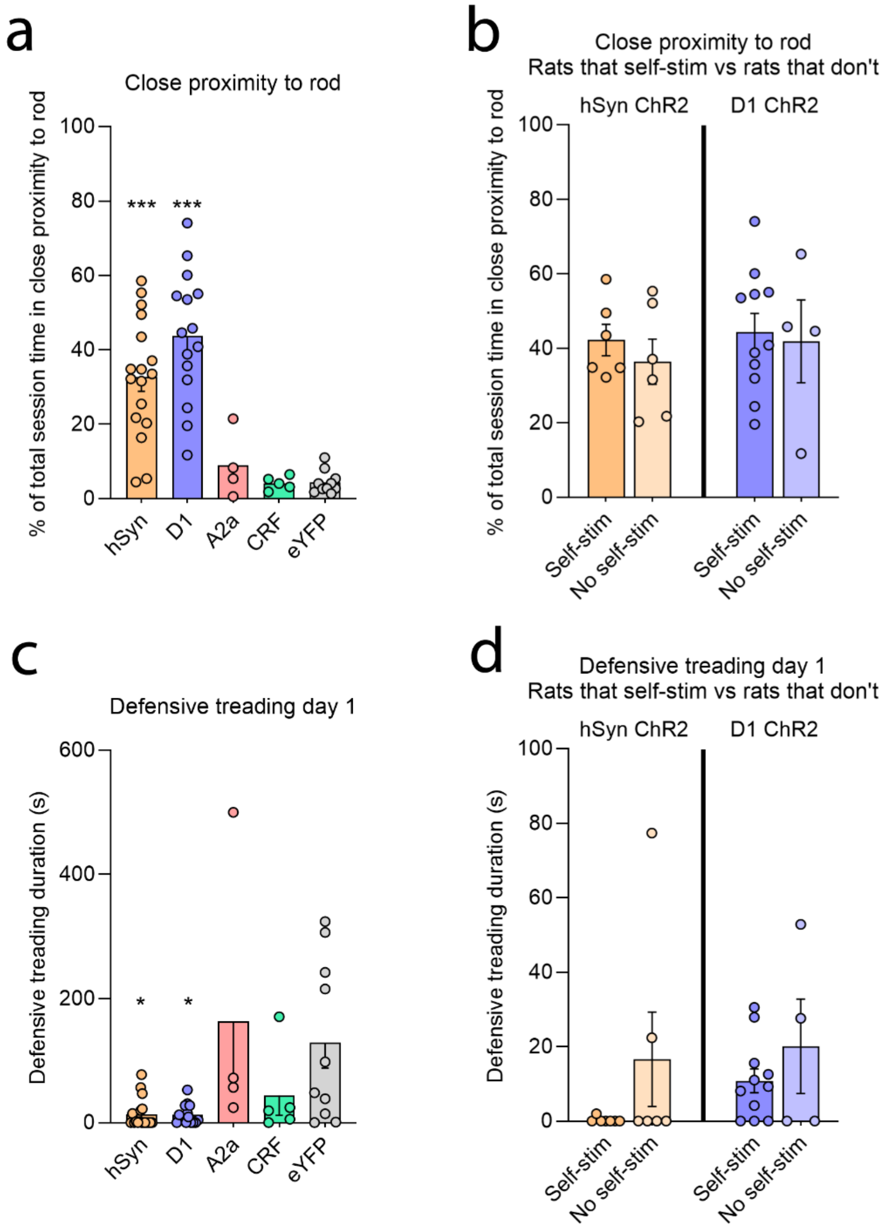

Supplementary Figure 2. a. hSyn ChR2 ($N = 17$) and D1 ChR2 rats ($N = 15$) spent a much higher percentage of time remaining in close < 2 cm proximity to the shock rod, compared to eYFP control ($N = 10$), A2(D2) ChR2 ($N = 4$), or CRF ChR2 rats ($N = 5$). **b.** In both hSyn ChR2 and D1 ChR2 groups, individuals that self-stimulate (hSyn ChR2 $N = 6$, D1 ChR2 $N = 11$) and those that don't (hSyn ChR2 $N = 6$, D1 ChR2 $N = 4$) do not differ in percentage of time remaining in close <2 cm proximity to the shock rod. **c.** Control eYFP rats showed significant antipredator defensive burying toward the shock rod on day 1, but hSyn ChR2 rats and D1 ChR2 rats did not. **d.** Defensive treading by hSyn ChR2 and D1 ChR2

group: hSyn individuals that self-stimulate and those that don’t self-stimulate do not differ in defensive treading.

Supplementary Figure 3: Barrier test

Male and female hSyn ChR2rats did not differ from each other in number of shocked barrier crosses (male *N* = 11, 11.09 ±2.48; female *N* = 5, 13.6 ±2.87; $t_{14} = 0.6$, $p = 0.56$), nor did male and female D1 rats ChR2 (male *N* = 6, 7.5 ±2.03; female *N* = 7, 12 ±2.54; $t_{11} = 1.35$, $p = 0.2$), or male and female eYFP rats (male *N* = 2, 2.5 ±0.5; female *N* = 6, 1 ±0.37; $t_6 = 2.12$, $p = 0.08$).

Overall, in terms of percentage of crossings that culminated in shock, both hSyn ChR2 and D1 ChR2 rats had a considerably higher proportion of shocked crossings (hSyn ChR2 93% ±3%; D1 ChR2 74% ±6%) than control eYFP rats (20% ±4%) ($F_{2,31} = 43.22$, $p < 0.001$, hSyn ChR2 $p < 0.001$, D1 ChR2 $p < 0.001$). The percentage of crossings that culminated in shock was also slightly higher for hSyn ChR2 rats than D1 ChR2 rats ($p = 0.014$) (Figure 4c).

Male and female rats did not differ in percentage of crossings that culminated in shock for either hSyn ChR2 (male *N* = 9, 93% ±3%; female *N* = 5. 90% ±5%; $t_{12} = 0.54$, $p = 0.6$) or D1 ChR2 rats (male *N* = 6, 74% ±10%; female *N* = 7. 75% ±9%; $t_{11} = 0.43$, $p = 0.97$).

Self-stim vs .no-stim. The percentage of crosses that culminated in shock by hSyn ChR2 rats that did self-stimulate (77% ±12%) was if anything slightly lower than in hSyn ChR2 rats that failed to self-stimulate (91% ±4%; $t_{15} = 0.85$, $p = 0.41$). The percentage of crosses that culminated in shock did not significantly differ between D1 ChR2 rats that did self-stimulate (78% ±6%) and those that failed to self-stimulate (66% ±13%; $t_{11} = 0.91$, $p = 0.38$).

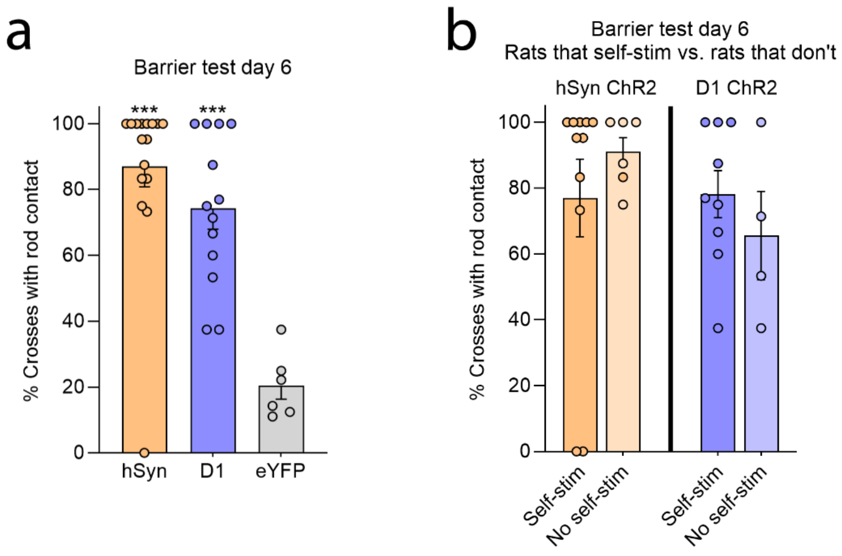

Supplementary Figure 3. a. Virtually all barrier crosses by hSyn ChR2 (*N* = 17) and D1 ChR2 (*N* = 13) rats culminated with a shock rod contact, whereas most crosses by eYFP rats (*N* = 6) did not result in contact. **b.** Separately shows the percentage of crosses that culminated in a shock rod contact for hSyn ChR2 and D1 ChR2 individuals that self-stimulated laser in the nose-poke/spout-touch task (hSyn ChR2 *N* = 11, D1 ChR2 *N* = 9) versus individuals that failed to self-stimulate (hSyn ChR2 *N* = 6, D1 ChR2 *N* = 4).

Supplementary Figure 4: Dummy rod

Comparing dummy rod to shock rod contacts, D1 ChR2 rats made over twice as many oral contacts with mouth, tongue or teeth on the shock rod (20.33 ±2.4) than on the dummy rod (8.33 ± 0.33) (t_2 = 5.77, p = 0.03) (Figure 5f). D1 ChR2 rats also more quickly returned to the shock rod after an oral contact to immediately re-initiate another chew contact within 1 – 3 sec after a flinch withdrawal (t_3 = 1.75, p = 0.18), whereas on the dummy rod they typically waited >10 sec to reinitiate chew contact after a pause (t_3 = 3.67, p = 0.034) (Figure 5e). Similarly, hSyn rats more quickly reinitiated a new paw touch contact <1 sec on the shock rod after a paw withdrawal (t_4 = 2.45, p = 0.07), whereas they took >10 sec to reinitiate paw contact on the dummy rod (t_4 = 1.5, p = 0.21) (Figure 5d).

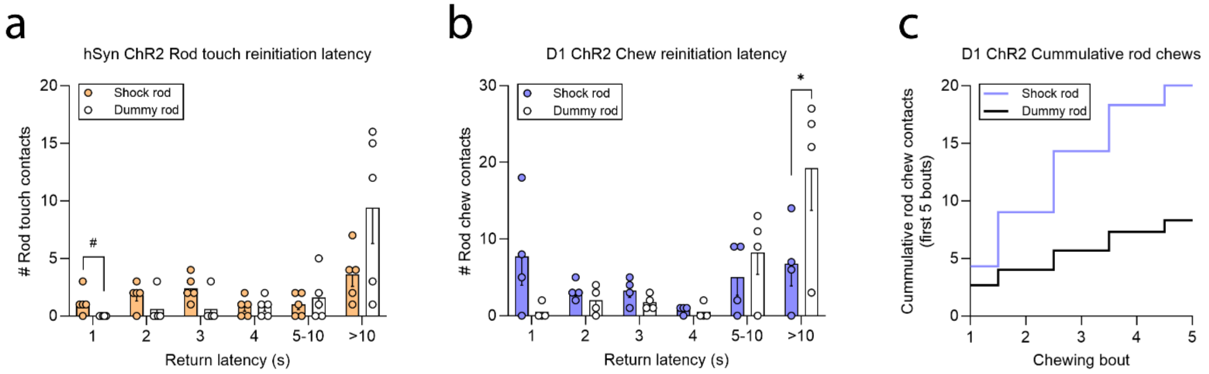

Supplementary Figure 4. a. hSyn ChR2 (N = 5) rats were slightly quicker to return to touch the shock rod than to the dummy rod after initially breaking a contact. **b.** D1 ChR2 (N = 4) rats were similarly quicker to return to oral contact on the shock rod than to the dummy rod after initially breaking contact. **c.** D1 ChR2 rats spent more time chewing on the shock rod than on the dummy rod.

Supplementary Note 3: Auditory CS+

hSyn ChR2. In particular, female hSyn ChR2 rats (19.33 ±2.19, $N = 3$) made more total responses on the CS+ day than male rats (9.2 ±1.16, $N = 5$; $t_6 = 4.56$, $p = 0.004$), but not did not differ on the CS- day ($t_6 = 2.08$, $p = 0.08$), and females also made more nosepokes on the CS+ day than they did on the CS- day ($t_2 = 4.6$, $p = 0.04$), while male rats did not differ between the two days ($t_4 = 0.91$, $p = 0.041$), suggesting that hSyn ChR2 females were more motivationally activated by the CS+ than male hSyn ChR2 rats. Overall, the higher total number of nosepokes on the CS+ day was due chiefly to more nosepokes into the inactive porthole on the day they heard the CS+ (6.5 ±1.21) than on the CS- day (3.63 ±0.56) ($t_7 = 2.31$, $p = 0.05$).

D1 ChR2. Again, this was especially pronounced in females ($N = 5$), which made 38.2 ±8.39 nosepokes on CS+ day but only 17.2 ±5.09 responses on the CS- day, whereas the male D1 ChR2 rat in this group made 17 nosepokes on the CS+ day, and 16 on CS- day. And again, D1 ChR2 rats appeared to generalize across the two portholes, making more nosepokes into both the CS+ earning porthole and inactive portholes on the CS+ day (CS+ porthole = 14.67 ±4.02; inactive porthole = 20 ±6.3) compared to the two portholes available on the CS- day (CS- porthole = 7.33 ±1.05; inactive porthole = 9.7 ±3.28), and there was no difference between portholes on either day (CS+ day: $t_5 = 1.85$, $p = 0.12$; CS- day: $t_5 = 2$, $p = 0.12$)

Control eYFP. Female ($N = 3$) and male rats ($N = 3$) did not differ in number of responses made on either CS+ ($t_4 = 1.47$, $p = 0.21$) or CS- day ($t_4 = 0.69$, $p = 0.53$). Control eYFP rats also did not discriminate between active and inactive portholes on either the CS+ day (active: 5 ±0.78, inactive: 4.33 ±0.67; $t_5 = 1.35$, $p = 0.24$) or CS- day (active: 5.5 ±0.85, inactive: 7.83 ±2.44; $t_5 = 1.32$, $p = 0.24$)

Supplementary Figure 5: Olfactory CS+

A naïve control group of rats that did not receive surgery or laser was found to have a preexisting sensory preference for vanilla (591.38 sec ±18.11) over orange (308.63 sec ±18.11; $t_7 = 7.81$, $p < 0.001$) (Figure 7d). Given that potential pre-existing bias against orange, we further focused on whether laser/shock rod pairings with orange was sufficient to reverse a potential orange aversion. The answer was yes: hSyn ChR2 rats spent 150% more time in the shock-associated orange side when it was their CS+ (451.4 s ±15.01) than naïve control rats spent with orange scent (308.63 sec ±18.11) ($t_{10.91} = 6.07$, $p < 0.001$). Likewise, D1 ChR2 rats spent >150% more time with the orange when it was their CS+ scent (504 sec ±37.03) than naïve control rats spent with orange ($t_{10} = 5.41$, $p < 0.001$), suggesting that the conditioned reinforcing properties of the orange CS+ reduced the natural aversiveness of the orange scent.

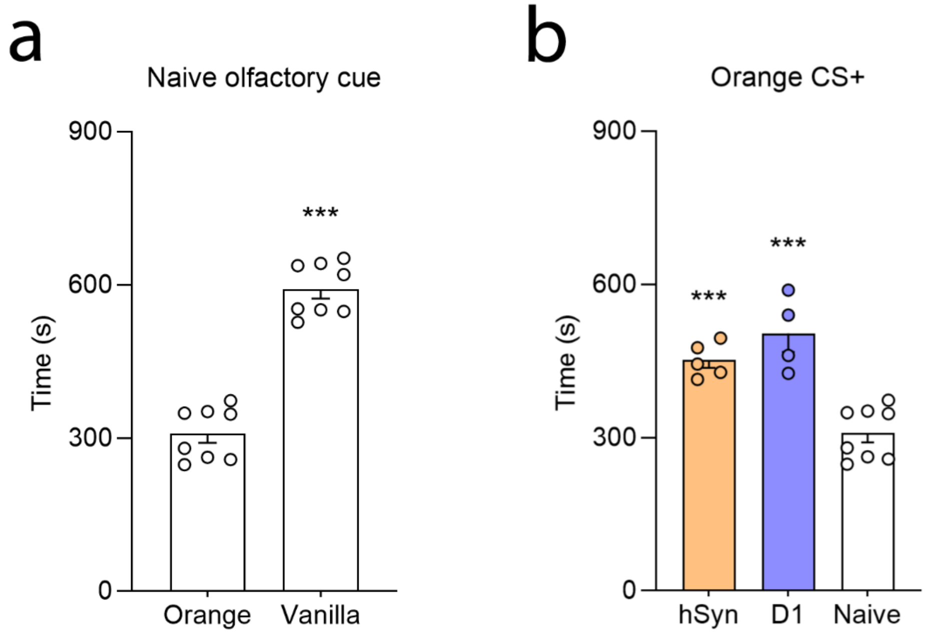

Supplementary Figure 5. a. Naïve unoperated rats ($N = 8$) showed a natural preference for vanilla scent over orange scent, **b.** but this naïve preference was abolished for D1 ChR2 ($N = 4$) or hSyn ChR2 rats ($N = 5$) if orange was paired with shock rod as their contextual CS+.

Supplementary Figure 6: RNAScope® Fluorescence In Situ Hybridization.
It seemed important to know for D1 ChR2 rats, which showed shock rod attraction, whether Cre was specifically expressed in CeA neurons that had D1 dopamine receptors. Colocalization of Cre and D1 RNA in infected CeA neurons was verified with fluorescence in situ hybridization following previously described procedures[4]. We were also interested in assessing whether these same neurons also had colocalization of Crf RNA. Brains from D1-Cre+ rats (N = 1 male, N = 1 female) were rapidly dissected and flash frozen in dry ice. The brains were then equilibrized for 1 hour in a Leica cryostat and sectioned into 17μm slices that included the CeA. Slices were thaw mounted on Superfrost plus slides (Fischer) and stored at -80°C. The slides were then fixed in 10% neutral buffered formalin for 30 min at 4°C. The slides were dehydrated for 5 min in 50% ethanol, 5 min in 70% ethanol, 5 min in 100% ethanol, and again for 5 min in 100% ethanol. The slides were dried for 20 min at room temperature, and a hydrophobic barrier was applied around the sections. The slides were then stored overnight in 5x saline-sodium citrate buffer. On the next day, the slides were washed twice with 1x wash buffer for 2 min at room temperature. Sections then underwent amplification at 40°C which consisted of 150 μL Amp 1 for 30 min, 150 μL Amp 2 for 30 min, and 150 μL Amp 3 for 15 min. Each amplification step was followed with two 2 min washes in wash buffer at room temperature. The slides were incubated in 150 μL in ACD RNAscope Probe iCre (catalogue # 423321), Rn-Drd1a-C2 (catalogue # 317031-C2), or Rn-Crf-C3 (catalogue # 318931-C3) for 15 min at 40°C, washed twice in wash buffer for 2 min at room temperature, incubated in 200 μL diluted Opal (520, 570, or 690) for 30 min at 40°C, washed twice in wash buffer for 2 min at room temperature, incubated in 150 μL HRP Blocker for 15 min at 40°C, and washed twice in wash buffer for 2 min at room temperature. The slides were then incubated in a DAPI-containing solution for 30 sec at room temperature, coverslipped with ProLong Gold Antifade Mountant, and then stored at 4°C. Images were taken at 40x magnification using a Leica epifluorescent microscope. Cells containing Cre, D1, or Crf messenger RNA were manually counted in 100 x 100 x 17 μm volume core samples in the CeA.

Cre mRNA and D1 mRNA were observed to be colocalized together in the same CeA neurons in D1 ChR2 slices, amounting to approximately 40% of all CeA neurons counted (5.33 ±1.45 co-labeled neurons per 100 x 100 x 17 μm volume sample). Remaining CeA neurons contained neither *Cre* mRNA nor D1 mRNA. There were no neurons observed expressing Cre but not D1 mRNAs, and none expressing D1 but not *Cre* mRNAs. Anatomically, *Cre*+ and D1+ co-expressing neurons were most densely concentrated in central and lateral subregions of CeA. All Cre+ and D1+ co-expressing neurons also contained *Crh* messenger RNAs.

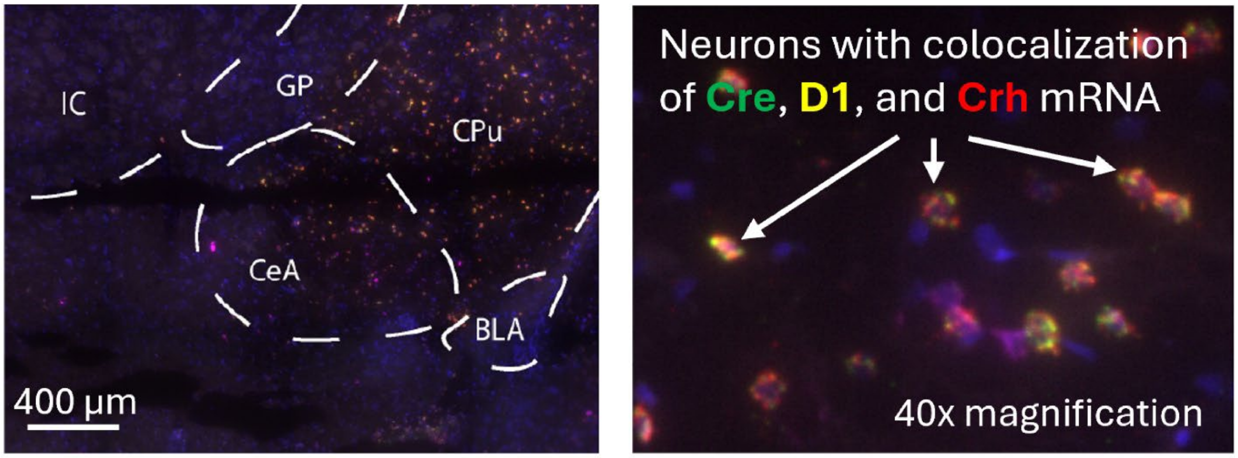

Supplementary Figure 6. mRNA Cre and D1 colocalization. D1 ChR2 photomicrographs show messenger RNA expression revealed by in situ hybridization. It reveals colocalization of Cre mRNA (green), D1 mRNA (yellow), and *Crh* mRNA (red) in the same CeA neurons of a D1 ChR2 brain.

Supplementary Note 4: Limitations

Our study has several limitations that future research could redress. We ran shock rod tests for all rats before self-stimulation tests, so as to not transfer any positive laser valence from self-stimulation procedures to the shock rod. This allowed us identify that pure shock rod attraction existed robustly for most individuals, even without a history of self-stimulation. However, future studies could vary the order of tests across individuals to assess if order matters. Another limitation is that our dummy rod procedure was not identical to our shock rod procedure here, although both were identical in an earlier study[1]. Here, we ran a single laser day for dummy rod, and a single no laser day in counterbalanced order, after one habituation session, to assess if laser pairing would elevate dummy rod approach over no-laser (it did not). Future studies might employ both dummy rod procedures to further assess the role of laser-seeking in rod attraction.

1. Warlow, S. M., Naffziger, E. E. & Berridge, K. C. The central amygdala recruits mesocorticolimbic circuitry for pursuit of reward or pain. *Nat Commun* **11**, 1–15 (2020).

2. Kim, J., Zhang, X., Muralidhar, S., LeBlanc, S. A. & Tonegawa, S. Basolateral to central amygdala neural circuits for appetitive behaviors. *Neuron* **93**, 1464-1479.e5 (2017).

3. Ponserre, M., Fermani, F., Gaitanos, L. & Klein, R. Encoding of environmental cues in central amygdala neurons during foraging. *Journal of Neuroscience* **42**, 3783–3796 (2022).

4. Baumgartner, H. M., Schulkin, J. & Berridge, K. C. Activating corticotropin-releasing factor systems in the nucleus accumbens, amygdala, and bed nucleus of stria terminalis: Incentive motivation or aversive motivation? *Biol Psychiatry* **89**, 1162–1175 (2021).
